# The Role of Calcium-Permeable AMPARs in Long-Term Potentiation at Principal Neurons in the Rodent Hippocampus

**DOI:** 10.3389/fnsyn.2018.00042

**Published:** 2018-11-22

**Authors:** Pojeong Park, Heather Kang, Thomas M. Sanderson, Zuner A. Bortolotto, John Georgiou, Min Zhuo, Bong-Kiun Kaang, Graham L. Collingridge

**Affiliations:** ^1^Department of Biological Sciences and Brain and Cognitive Sciences, College of Natural Sciences, Seoul National University, Seoul, South Korea; ^2^Department of Physiology, Faculty of Medicine, University of Toronto, Toronto, ON, Canada; ^3^Lunenfeld-Tanenbaum Research Institute, Mount Sinai Hospital, Toronto, ON, Canada; ^4^Centre for Synaptic Plasticity, School of Physiology, Pharmacology and Neuroscience, University of Bristol, Bristol, United Kingdom

**Keywords:** NMDA receptor, long-term potentiation, hippocampus, calcium-permeable AMPA receptor, PKA

## Abstract

Long-term potentiation (LTP) at hippocampal CA1 synapses is classically triggered by the synaptic activation of NMDA receptors (NMDARs). More recently, it has been shown that calcium-permeable (CP) AMPA receptors (AMPARs) can also trigger synaptic plasticity at these synapses. Here, we review this literature with a focus on recent evidence that CP-AMPARs are critical for the induction of the protein kinase A (PKA)- and protein synthesis-dependent component of LTP.

## Introduction

Long-term potentiation (LTP) is the most intensively studied type of synaptic plasticity in the vertebrate central nervous system (CNS), driven by the widely-held view that the mechanism is engaged by, and is critical for, learning and memory processes (Bliss and Collingridge, 1993). LTP has been most extensively studied at the Schaffer collateral-commissural pathway, a monosynaptic connection between CA3 and CA1 pyramidal neurons of the hippocampus. The principles uncovered by studying these synapses have been generalized to many, but not all, excitatory synapses in the CNS. It is established that there are several distinct forms of LTP that co-exist over the first few hours following its induction (Park et al., 2014). The first to be observed is termed short-term potentiation (STP), which is triggered by high frequency stimulation (HFS) and decays in an activity-dependent manner (Volianskis and Jensen, 2003). Without post-induction stimulation, STP can last for, at least, many hours. STP is expressed by an increase in the probability of neurotransmitter release, P(r). Multiple, mechanistically distinct forms of LTP can also be induced, which are often termed LTP1, LTP2 and LTP3 (see Abraham and Otani, 1991; Bliss and Collingridge, 1993; Reymann and Frey, 2007 for reviews). Here, we adopt the following nomenclature: LTP1 (also referred to as early-LTP or E-LTP) can be triggered by a single episode of HFS, such as a tetanus or theta burst stimulation (TBS) protocol. It is also commonly induced by multiple episodes of HFS or by “pairing” protocols. LTP1 can last for several hours, at least. In adult rodents, it requires activation of Ca^2+^/calmodulin-dependent protein kinase II (CaMKII) but is independent of both PKA and protein synthesis. There is considerable evidence that it is expressed by an increase in the number of AMPA receptors (AMPARs) inserted into the postsynaptic membrane. LTP2 (also referred to as late phase-LTP or L-LTP) normally requires multiple episodes of HFS (e.g., tetani or TBS) for its induction; critically these episodes need to be appropriately spaced in time, typically in the order of 10 min. Since these spaced protocols will also induce LTP1, the resulting potentiation is a composite of LTP1 and LTP2, initially at least. LTP2 can last many hours *in vitro* and, most probably, is associated with synaptic growth with corresponding pre- and postsynaptic alterations. Unlike LTP1, LTP2 requires activation of protein kinase A (PKA) and *de novo* protein synthesis, but does not involve transcription. LTP3 requires transcription and is not considered further in the present article. For a recent review of these three types of synaptic plasticity the reader is referred to Bliss et al. (2018).

In terms of the induction trigger, all these forms of synaptic plasticity ordinarily require the synaptic activation of NMDA receptors (NMDARs). However, there are some differences in their NMDAR subunit dependence. LTP1 is induced primarily by the activation of triheteromeric assemblies of GluN1, GluN2A and GluN2B whereas STP has a component that requires activation of GluN2B and GluN2D (Volianskis et al., 2013a). Since the NMDAR is subject to a highly voltage-dependent block by Mg^2+^ ions, which is transiently alleviated during HFS (Herron et al., 1986), the NMDAR confers Hebbian-like properties on LTP. Under certain circumstances, metabotropic glutamate receptors (mGluRs) can serve as co-triggers for the induction of NMDAR-dependent LTP (Bashir et al., 1993; Bortolotto et al., 1994), possibly by facilitating the activation of NMDARs (Tigaret et al., 2016).

**Figure 1:**
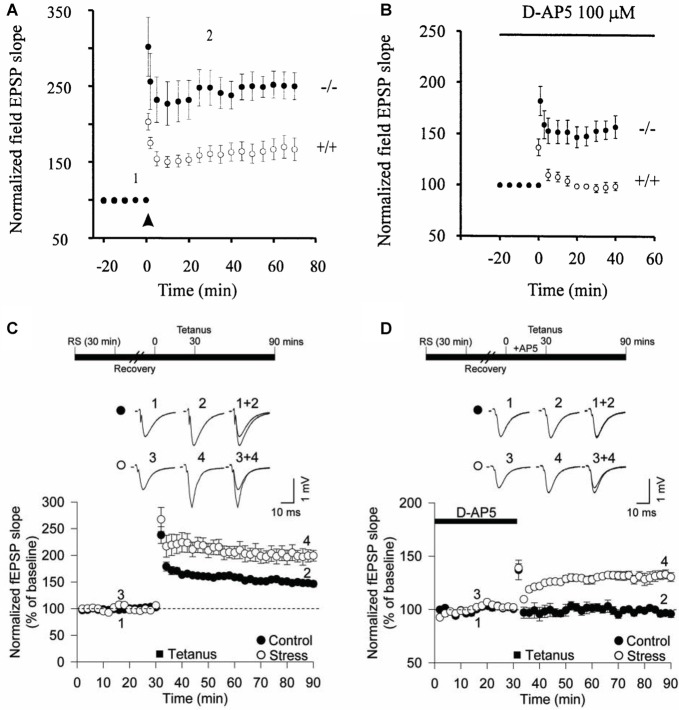
Calcium-permeable (CP)-AMPA receptors (AMPARs) can trigger long-term potentiation (LTP) at CA1 synapses. **(A)** Larger LTP in GluA2^−/–^ mice compared with wild-type littermates. **(B)** NMDA receptors (NMDARs)-independent LTP in the GluA2^−/–^ mice. **(C)** Acute restraint stress facilitates LTP. **(D)** Acute restraint stress enables NMDAR-independent LTP. Panels **(A,B)** from Jia et al. (1996) and **(C,D)** from Whitehead et al. (2013).

The first suggestion that calcium-permeable (CP)-AMPARs could be triggers for LTP at CA1 synapses was the finding that, in mice engineered to lack the GluA2 subunit, LTP could be induced when NMDARs were blocked (Jia et al., 1996; Figures 1A,B). Since GluA2-lacking receptors are calcium permeable (Burnashev et al., 1992), this suggested that CP-AMPARs may be alternative triggers for LTP at CA1 synapses. This form of LTP probably involves a postsynaptic change in the number or conductance properties of AMPARs (Mainen et al., 1998). In support of the idea that CP-AMPARs can trigger LTP at these synapses, transfection of unedited GluA2 into CA1 pyramidal neurons enabled NMDAR-independent LTP to be induced (Okada et al., 2001).

Interestingly, in the GluA2 KO mouse in the absence of an NMDAR antagonist, both the CP-AMPAR and NMDAR-dependent forms of LTP were additive, suggesting two distinct forms of LTP can co-exist at CA1 synapses (Jia et al., 1996). These pioneering experiments did not, however, address whether CP-AMPARs are engaged during synaptic plasticity in wild type animals. The lack of GluA2 results in the formation of aberrant glutamate receptor complexes involving GluA1 and GluA3 subunits as well as an increase in the number of GluA1 and GluA3 homomeric receptors (Sans et al., 2003). Furthermore, in both a different GluA2 global KO and in a forebrain-specific GluA2 KO, the LTP appeared normal (Shimshek et al., 2006), however there was enhanced STP in the conditional GluA2 KO. Therefore, the significance of CP-AMPARs as triggers for synaptic plasticity was unclear.

**Figure 2:**
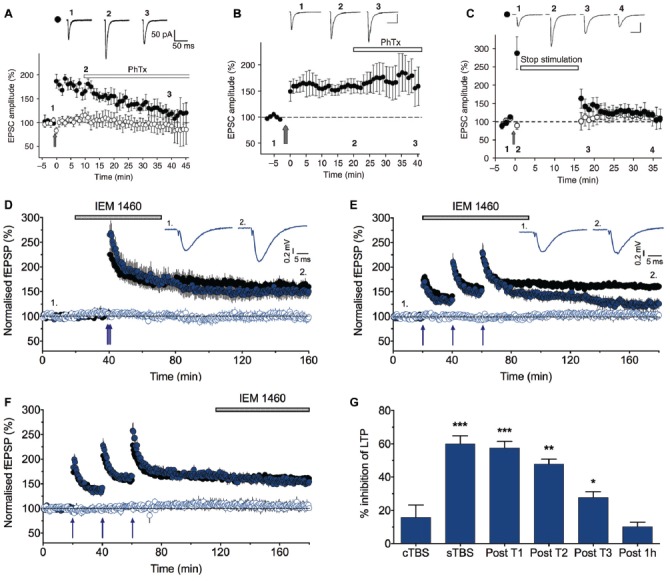
LTP2, but not LTP1, is sensitive to CP-AMPAR blockers. **(A)** Philanthotoxin (PhTx) reverses the potentiation when applied starting 10 min after pairing-induced LTP.** (B)** PhTx has no effect when applied starting 20 min after the induction of LTP.** (C)** LTP is not induced when baseline stimulation is paused for 15 min following pairing (from Plant et al., 2006). **(D)** IEM 1460 (30 μM) has no effect on LTP induced by compressed protocol (cTBS; *n* = 8 and 6 for vehicle and IEM experiments, respectively). **(E)** IEM 1460 applied immediately following the first theta burst stimulation (TBS) inhibits LTP. **(F)** IEM 1460 applied 1 h following the last spaced protocol (sTBS) has no effect on LTP. **(G)** Summary data (*n* = 6–10 for IEM experiments, *n* = 21 for interleaved controls). Each graph plots the mean ± SEM normalized fEPSP slope for vehicle-treated (black) and interleaved drug-treated (color) slices. The open symbols are the corresponding control (no TBS) inputs. Representative traces (average of five successive recordings) are shown for the times indicated by numbers. *p < 0.05, **p < 0.01 and ***p < 0.001 vs. control. Reproduced from Park et al. (2016).

A clue to a physiological role for these receptors in wild type animals was the finding that inhibitors of CP-AMPARs could largely reverse LTP if applied shortly after the induction of LTP (Figure 2A). However, if the application of these inhibitors was delayed for 30 min or so, they were no longer effective (Figure 2B). The implication from these observations is that there is a short time window following the triggering of LTP where CP-AMPARs may be required for the full expression of LTP. It was also found that if baseline stimulation was paused during this same time window following the induction of LTP, then LTP was not fully expressed (Figure 2C). This suggested that low frequency synaptic activation of CP-AMPARs was required to fully sustain LTP. The obvious inference was that low frequency stimulation triggers Ca^2+^ entry through CP-AMPARs and that this Ca^2+^ signal is required for the full expression of LTP. However, two other labs using similar methods found no evidence for a role of CP-AMPARs in LTP at CA1 synapses (Adesnik and Nicoll, 2007; Gray et al., 2007). Another LTP controversy was brewing.

The reason for these differences was not readily apparent at the time. But further evidence that CP-AMPARs can contribute, under certain circumstances, to LTP at these synapses was the finding that these receptors enabled an NMDAR-independent form of LTP following brief exposure of the animals to acute restraint stress (Whitehead et al., 2013). Indeed, the effect of brief stress, or exposure to glucocorticoids, resulted in a situation highly reminiscent of the GluA2 KO mouse (Figures 1C,D). So what was clear from these earlier studies is that CP-AMPARs can, but do not necessarily, contribute to LTP at CA1 synapses onto principal neurons. We became interested, therefore, in establishing the physiological conditions that determined whether CP-AMPARs participate in LTP at these synapses.

## Pharmacological Evidence That CP-AMPARs Are Required for LTP2 but Not LTP1

We re-investigated the role of CP-AMPARs in LTP by comparing the effect of two types of induction protocols (Park et al., 2016). We delivered three episodes of TBS, with each episode comprised of five bursts (each of five shocks at 100 Hz) with an inter-burst frequency of 5 Hz (total 75 stimuli). Notably, the only difference between the two interleaved protocols was the inter-episode interval. We either used a compressed protocol (cTBS) where the inter-episode interval was 10 s or a spaced protocol (sTBS) where the inter-episode interval was in the order of minutes (typically 10 or 20 min). cTBS led to an LTP that is, after STP had decayed, exclusively LTP1. In contrast, sTBS induced an LTP that was a mixture of LTP1 and LTP2. In agreement with previous work (Matthies and Reymann, 1993; Huang and Kandel, 1994), LTP1 was resistant to inhibitors of PKA while LTP2 was sensitive to these inhibitors. We found a similar differential sensitivity to inhibitors of CP-AMPARs (Figures 2D–G). Thus, IEM 1460 (IEM; Samoilova et al., 1999), philanthotoxin 433, or 1-naphthyl acetyl spermine (NASPM) had no effect on LTP induced by a cTBS (i.e., LTP1) but inhibited a component of sLTP (presumably LTP2). Consistent with the previous study (Plant et al., 2006), IEM was still effective if applied shortly after the TBS but not if applied 1 h following the TBS (Figures 2E–G), confirming a transient time window for the involvement of CP-AMPARs.

## Stimulation Post TBS Is Required for LTP2

**Figure 3:**
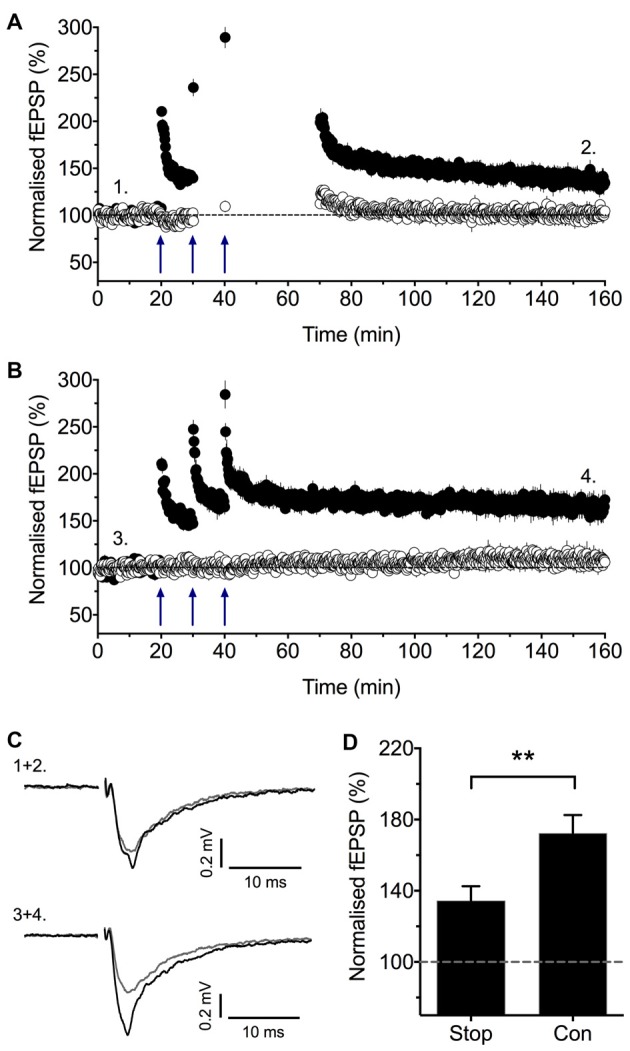
Stimulation post-TBS is required for LTP2. **(A)** Baseline stimulation was paused following the second and third sTBS episodes (apart from an initial stimulation to assess short-term potentiation, STP) for nine experiments. **(B)** Interleaved control experiments where there was no pause in stimulation (*n* = 7). **(C)** Representative traces (at the times indicated by numbers). **(D)** Quantification of these experiments (2 h post TBS). Reproduced from Park et al. (2016). **p < 0.01 vs. control.

Consistent with the earlier report (Plant et al., 2006) that stimulation is required post induction for the full expression of LTP, we observed that if we stopped stimulation following the sTBS protocol then LTP was reduced (Figure 3). We interpreted this result to mean that, for a short time window after the synaptic activation of NMDARs, low frequency stimulation is required to induce LTP2 and, consequently, when stimulation is paused then only LTP1 is observed. In conclusion, there exists a critical period following sTBS that requires low frequency synaptic activation and the synaptic expression of CP-AMPARs to generate the PKA- and protein synthesis-dependent form of LTP (i.e., LTP2). The simplest explanation is that low frequency stimulation drives Ca^2+^ through CP-AMPARs and that this Ca^2+^ signal is required for the *de novo* protein synthesis.

One difference between these observations and the previous study that revealed a role for CP-AMPARs (Plant et al., 2006) is in the induction protocols that were used to generate LTP. The earlier study did not use a spaced induction protocol and so, according to our recent results, should have induced only LTP1. In which case, the LTP would have been insensitive to blockers of CP-AMPARs and resistant to a pause in stimulation, just as described in two other studies (Adesnik and Nicoll, 2007; Gray et al., 2007). However, there may be metaplastic effects that can prime for LTP2. One likely factor is the level of stress that an animal experiences. As described above, a brief period of restraint stress leads to the PKA-dependent insertion of CP-AMPARs into the synaptic membrane (Whitehead et al., 2013). Therefore, stress may lead to the synaptic insertion of CP-AMPARs such that a spaced induction protocol is no longer required to induce LTP2. In conclusion, while stress and probably other factors that activate PKA, may prime for LTP2, a spaced induction protocol is typically required for its expression under most physiological conditions.

In conclusion, our pharmacological experiments have clearly identified two forms of LTP that co-exist at CA1 synapses that can be distinguished on the basis to their sensitivity to inhibitors of CP-AMPARs. LTP1 does not require their activation, whereas LTP2 does require CP-AMPARs. Furthermore, LTP2 requires baseline stimulation following TBS. The reports of Adesnik and Nicoll (2007) and Gray et al. (2007) are not inconsistent with this conclusion, since their protocols would have only induced LTP1.

## Physiological Evidence That CP-AMPARs Are Required for LTP2 but Not LTP1

Rather than to rely on pharmacological evidence alone, we also probed for the synaptic insertion of CP-AMPARs by measuring synaptic AMPAR rectification. GluA2-lacking AMPARs are inwardly rectifying due to an endogenous voltage-dependent block by spermine (Donevan and Rogawski, 1995; Isa et al., 1995) and so we measured their rectification index (RI) shortly after inducing LTP (Plant et al., 2006; Adesnik and Nicoll, 2007) with cTBS and sTBS protocols. We found no change in the RI following cTBS but a significant increase in rectification following sTBS (Park et al., 2016). These observations are consistent with the synaptic insertion of CP-AMPARs into a background of calcium-impermeable (CI) AMPARs.

The PKA-dependent component of LTP (i.e., LTP2) can also be induced by a single episode of TBS delivered under conditions where cAMP generation is enhanced, such as in the presence of the phosphodiesterase 4 (PDE4) inhibitor rolipram (Barad et al., 1998). We found that the rolipram-enhanced LTP was due to the insertion of CP-AMPARs as probed either with IEM 1460 or using RI measurements (Park et al., 2016).

## A Model for the Induction of LTP at CA1 Synapses

**Figure 4:**
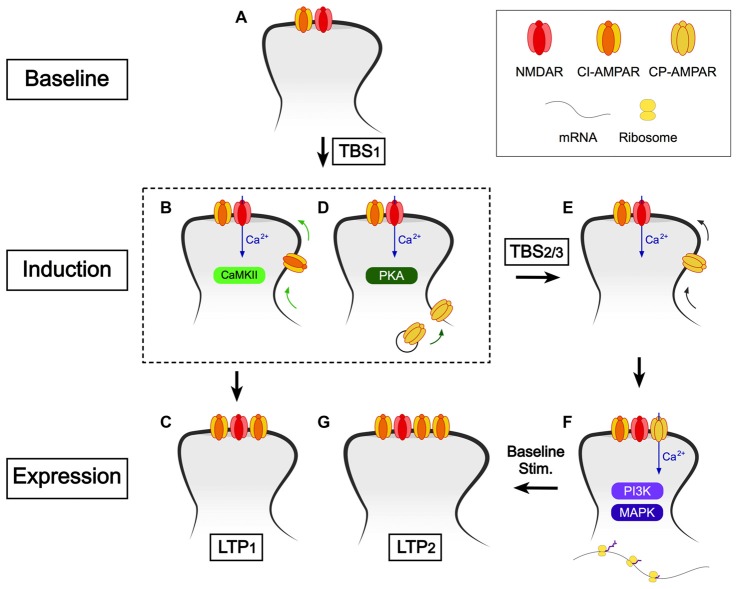
A scheme to explain how CP-AMPARs may trigger LTP2. **(A)** Baseline conditions. The synaptic complement comprises NMDARs (predominantly GluN1/GluN2A/GluN2B) and calcium impermeable (CI)-AMPARs (note only one of each receptor type is shown for simplicity). **(B,C)** The first TBS (or cTBS, cHFS, etc.) induces LTP1 via activation of CaMKII and involves the synaptic insertion of additional CI-AMPARs. **(D)** The first TBS also drives CP-AMPARs into the perisynaptic plasma membrane, via a pathway involving protein kinase A (PKA). **(E)** Additional TBS activates NMDARs to drive CP-AMPARs from the perisynaptic to the synaptic membrane. **(F)** Baseline stimulation leads to Ca^2+^ entry via CP-AMPARs, which triggers *de novo* protein synthesis (via PI3K and MAPK). **(G)** Consequently, what follows is spine growth and the incorporation of additional CI-AMPARs. CP-AMPARs are removed from the synapse around the same time.

The mechanism of induction of LTP is rather more complex than originally proposed (Collingridge, 1985). In Figure 4, we present a model based on our current understanding, which elaborates upon our earlier model (Park et al., 2016). For simplicity we have omitted STP. A compressed induction protocol (e.g., cTBS) induces only LTP1, which may involve the CaMKII-dependent insertion of more AMPARs (Hayashi et al., 2000; Lu et al., 2001; Pickard et al., 2001). It also primes for LTP2 by activating PKA (Frey et al., 1993; Matthies and Reymann, 1993), which drives CP-AMPARs into the perisynaptic plasma membrane, via a process involving phosphorylation of S845 of GluA1 (He et al., 2009). We propose that if a second TBS occurs while these CP-AMPARs are on the perisynaptic plasma membrane then they are driven into the synapse, from where they can be activated by low frequency stimulation to trigger LTP2. If, however, there is no subsequent TBS during this time window, which is of the order of minutes, then the CP-AMPARs are removed from the perisynaptic plasma membrane, presumably by endocytosis. In this model, the period of time during which the second and third TBS can elicit LTP2 is determined by the dwell time of CP-AMPARs on the plasma membrane. If the interval is too short (i.e., < ~1 min) the CP-AMPARs would not have had time to traffic to the perisynaptic membrane. If the interval is too long (>~1 h) then the CP-AMPARs would have been removed from the perisynaptic membrane.

It should also be noted that our findings could be explained by the activation of CP-AMPARs that are already present at the synaptic membrane, rather than the need for their physical trafficking (Rozov et al., 2012). For example, the first TBS might phosphorylate CP-AMPARs and that this is a pre-requisite for the subsequent TBS to activate these receptors. However, we have based our model on the extensive literature that PKA drives the insertion of GluA1, via phosphorylation of S845, onto the plasma membrane (Man et al., 2007) from where it is trafficked from perisynaptic/extrasynaptic locations to synaptic sites (e.g., He et al., 2009; Makino and Malinow, 2009; Yang et al., 2010). Both models assume that CP-AMPARs are present for periods of time on the plasma membrane of dendritic spines of CA1 neurons, for which considerable evidence exists (e.g., Mattison et al., 2014).

The concept of the first TBS providing a priming for the subsequent TBS can be considered as a form of metaplasticity. Related to this, it has been shown that prior synaptic activity can lead to altered sensitivity to mGluR antagonists and enhanced LTP, via the activation of mGluRs (Bortolotto et al., 1994; Cohen et al., 1998). This additional form of LTP also requires *de novo* protein synthesis, and so is likely to represent LTP2 (Raymond et al., 2000). The priming, by the first episode of synaptic activity, is mediated by mGlu5 receptors (Bortolotto et al., 2005). Thus, there are different ways by which the protein synthesis machinery can be engaged to trigger LTP2.

Our new model raises several questions that require further experimentation:

1. What are the roles of NMDARs during the induction of LTP2? Are these only required during the first, priming TBS to activate PKA or are they also required during the subsequent TBS to, for example, drive the CP-AMPARs from the perisynaptic to the synaptic membrane? The latter could, in principle, not require activation of NMDARs since CP-AMPARs can trigger LTP in an NMDAR-independent manner (Jia et al., 1996; Whitehead et al., 2013).
2. What are the signaling mechanisms that drive CP-AMPARs from perisynaptic to synaptic sites, in response to additional bouts of TBS? There have been several suggestions, including CaMKII (Hayashi et al., 2000), CaMKI (Guire et al., 2008) and PKC (Yang et al., 2010).
3. Why is low frequency stimulation (i.e., basal synaptic transmission) required during the critical time window? What seems to be the simplest explanation is that low frequency stimulation drives Ca^2+^ through CP-AMPARs and that this specific Ca^2+^ source is required for LTP2 (Morita et al., 2014). Certain of the downstream signaling molecules that are involved in LTP triggered by the activation of CP-AMPARs have been identified by studying NMDAR-independent LTP in the GluA2 homozygous and heterozygous KO mouse. This LTP involves postsynaptic Ca^2+^, PI3K, MAPK and protein synthesis but not, unlike LTP1, CaMKII (Asrar et al., 2009). On the reasonable assumption that the same signaling processes are engaged by CP-AMPARs to trigger LTP2 in wild type animals then it seems likely that one or more components of this pathway specifically require the CP-AMPAR-generated Ca^2+^ signal. NMDAR activation alone (during sTBS) is insufficient to trigger LTP2 presumably due to different spatiotemporal properties of the two Ca^2+^ sources. In other words, CP-AMPARs may elevate Ca^2+^ within a microdomain that is specifically associated with the protein synthesis machinery. Alternatively, the activation of protein synthesis may also require a more prolonged Ca^2+^ signal, provided by baseline stimulation, rather than only the brief, but intense, Ca^2+^ signals during the episodes of TBS.
4. Why is the insertion of CP-AMPARs only transient and what does it trigger to sustain the potentiation? One may speculate that persistent insertion of CP-AMPARs could lead to neurotoxicity due to sustained synaptic Ca^2+^ entry (Noh et al., 2005; Dias et al., 2013). Since their dwell time on the plasma membrane is less than ~1 h, alternate means must sustain the potentiation. The most likely explanation is that CP-AMPARs trigger rapid synaptic growth, involving *de novo* protein synthesis, and that the enhanced synaptic response is maintained, at least in part, by the insertion of additional GluA2-containing, CI-AMPARs. Consistent with this idea, during glycine-induced LTP in cultured neurons, CP-AMPARs are necessary for spine enlargement via regulation of actin polymerization (Fortin et al., 2010).
5. What are the mechanisms that limit the synaptic expression of CP-AMPARs under basal conditions and how are these regulated during sLTP? The first clue to a mechanism was the finding that over-expression of PICK1 leads to the synaptic insertion of CP-AMPARs in a PKC and CaMKII-dependent manner (Terashima et al., 2004). A putative mechanism involves the binding of PICK1 to GluA2 to internalize CI-AMPARs and so paving the way for the insertion of CP-AMPARs. In addition to PICK1, CP-AMPAR plasticity involves NSF (Gardner et al., 2005); a protein that binds GluA2 to stabilize synaptic AMPARs (Nishimune et al., 1998). Indeed, there is direct evidence that the switch from CP-AMPARs to CI-AMPARs following LTP involves GluA2 interactions with both NSF and GRIP/PICK1 (Yang et al., 2010).
  Related to this, it was shown that during glycine-induced LTP in cultured hippocampal neurons there was rapid insertion of GluA1 but not GluA2, due to PICK1 retention of GluA2 at the endosomal membrane (Jaafari et al., 2012). This interaction is then disrupted following the activation of CP-AMPARs enabling the insertion of CI-AMPARs. This second step in the process resembles a form of synaptic plasticity first identified at cerebellar stellate cell synapses where repetitive activation of CP-AMPARs leads to their replacement by CI-AMPARs (Liu and Cull-Candy, 2000), a process that also involves PICK1 (Liu and Cull-Candy, 2005).
6. Why do three forms of long-lasting synaptic plasticity (STP, LTP1, LTP2) co-exist at CA1 synapses? Why STP exists is an easier topic to discuss. Since STP is expressed presynaptically (Davies et al., 1989), as an increase in P(r) (Volianskis and Jensen, 2003), its effects are additive to LTP1 and LTP2, which are expressed in part, if not exclusively, by postsynaptic alterations. Notably, STP and LTP1 differentially affect high frequency burst discharges, a major firing mode in the hippocampus. LTP scales all responses equally within a burst while STP alters the dynamic response within a burst, since it directly modulates P(r) (Volianskis et al., 2013b). The ability of STP to store information for variable periods of time until it is erased by activity could be important for certain forms of short-term memory, where information is stored until needed and then quickly forgotten.
  With respect to LTP1 and LTP2, these could underlie distinct forms of memory that are distinguished by the duration of the information encoded. Although both LTP1 and LTP2 can persist for many hours in slices it seems likely that in the living animal LTP2 will last for considerably longer. That is to say that a compressed episode of HFS (e.g., cTBS) triggers LTP1 that does not require protein synthesis and is expressed primarily, or exclusively, by changes in the number or conductance properties of AMPARs (Benke et al., 1998; Davies et al., 1989; Malinow and Malenka, 2002; Collingridge et al., 2004). In contrast, appropriately timed multiple trains induce LTP2 that requires protein synthesis to enable changes that are much more persistent. These likely involve structural modifications affecting both pre- and post-synaptic function. Indeed, there is considerable behavioral evidence that spaced training (that may be equated to spaced LTP induction protocols) leads, via a PKA-dependent process, to more persistent memories than massed training (which may be equated to compressed LTP induction protocols; Nonaka et al., 2017). Accordingly, the recruitment of CP-AMPARs into synaptic plasticity processes is likely to be crucial for some, but not all, forms of NMDAR-dependent learning and memory in the hippocampus (Wiltgen et al., 2010). The association between CP-AMPARs and both PKA and *de novo* protein synthesis suggest that they will be involved in the laying down of more salient memories, such as those linked to fear (Clem and Huganir, 2010).

## CP-AMPARs in Other Forms of Synaptic Plasticity at CA1 Synapses Onto Pyramidal Neurons

In addition to one type of LTP, discussed above, CP-AMPARs have also been implicated in the induction of NMDAR-dependent LTD at CA1 synapses (Sanderson et al., 2016). It was proposed that in LTD, the influx of Ca^2+^ through NMDARs recruits CP-AMPARs (GluA1 homomers phosphorylated on Ser845). Next, Ca^2+^ influx through both NMDARs and CP-AMPARs leads to activation of calcineurin and, in turn, to the internalization of both CP-AMPARs and CI-AMPARs, as well as spine shrinkage. This model, therefore, has several similarities to what we have proposed for LTP2. The direction of the alteration in synaptic efficacy (LTP vs. LTD) may be determined, at least in part, by the kinetics of the Ca^2+^ signals.

CP-AMPARs have also been shown to be involved in homeostatic scaling and related phenomena (e.g., Ju et al., 2004; Thiagarajan et al., 2005; Soares et al., 2013; Kim and Ziff, 2014; Kim et al., 2015; Sanderson et al., 2018). Here, the mechanisms appear to be very similar to those involved in LTP and LTD, further supporting the idea that homeostatic plasticity utilizes conventional synaptic plasticity mechanisms to provide more widespread balancing of synaptic weights.

## CP-AMPARs at Other Hippocampal Synapses

In this review, we have focussed on the role of CP-AMPARs at synapses made between CA3 and CA1 pyramidal neurons; the pathway which has been the most intensely investigated with respect to synaptic plasticity mechanisms. However, it is worth mentioning that excitatory synapses onto many classes of inhibitory interneurons within the hippocampal formation also involve CP-AMPARs (e.g., McBain and Dingledine, 1993; Topolnik et al., 2005). At these synapses, CP-AMPARs play critical roles in synaptic plasticity (Laezza et al., 1999; Toth et al., 2000; Ross and Soltesz, 2001; Lei and McBain, 2002; Oren et al., 2009; Croce et al., 2010; Szabo et al., 2012). Why there is a difference between pyramidal neurons and these interneurons is not known. However, it has been suggested this may relate to their firing patterns. For example, some interneurons lack NMDAR-dependent synaptic plasticity and so can follow high frequencies of stimulation (e.g., during theta and gamma rhythms) with a stable output but remain modifiable by virtue of CP-AMPAR-dependent synaptic plasticity (Szabo et al., 2012).

## CP-AMPARs During Development

We have focused on the role of CP-AMPARs at CA3-CA1 synapses in adult tissue. It should be pointed out, however, that early in development the situation is quite different. Whereas CP-AMPARs do not contribute to fast basal synaptic transmission in adults they participate in synaptic transmission in a subset of these synapses early in development, before around P7 (Stubblefield and Benke, 2010). These receptors may be removed during LTP-like activity since around this stage of development one form of LTP involves an increase in potency but a decrease in single channel conductance (Palmer et al., 2004); findings that are most simply explained by an exchange of high conductance CP-AMPARs for a greater number of CI-AMPARs. The idea of a subunit switch during development from CP-AMPARs to CI-AMPARs is also supported by gene expression studies (Pellegrini-Giampietro et al., 1992) and surface immunolabeling of cultured hippocampal neurons (Pickard et al., 2000).

Another study has found evidence for a role of CP-AMPARs in LTP at CA1 synapses that is developmentally regulated. In response to a single high frequency tetanus, GluA2-lacking AMPARs were required for the full expression of LTP in mice of 2-weeks and 8-weeks of age, but not in mice of 3- or 4-weeks of age (Lu et al., 2007). There was again a correlation between sensitivity of inhibitors of CP-AMPARs and the requirement for PKA. The protocol used in these studies is equivalent to a compressed one and would, according to our study (which was performed in rats of 3–12 weeks of age), have induced LTP that is totally independent of CP-AMPARs. However, the age of the animals used is probably relevant. We have reported previously that, in 2-week old rats, two forms of LTP can be induced by a tetanus and that one form involves a PKA-dependent mechanism (Wikström et al., 2003). However, why there is a re-appearance of this mechanism in 8-week old mice is less clear.

## CP-AMPARs in Disease Models

A role for CP-AMPARs in hypoxia/ischemeia is well established (e.g., Noh et al., 2005; Liu et al., 2006; Dias et al., 2013; Quintana et al., 2015). In addition, there is growing evidence for an involvement in other conditions, such as status epilepticus (Rajasekaran et al., 2012) and Alzheimer’s disease (AD). Interestingly, a very early change in a mouse model of AD (APPswe; PS1ΔE9 transgenic mice) is an enhanced LTP which may be due to the synaptic incorporation of CP-AMPARs (Megill et al., 2015). Related to this, it has been shown that oligomeric Aβ (1–42) when delivered intracellularly leads to extremely rapid synaptic insertion of CP-AMPARs, via a PKA-dependent mechanism (Whitcomb et al., 2015). Therefore, by analogy to NMDARs (Zhou and Sheng, 2013), CP-AMPARs are important for normal physiological function but their expression needs to be tightly regulated to prevent neurotoxicity and other pathological conditions.

## Concluding Remarks

Here we reviewed the role of CP-AMPARs in the induction of LTP at synapses onto pyramidal neurons in the hippocampus. We show that these receptors are critically involved in the PKA and protein synthesis dependent form of LTP. In addition to this role in LTP2, there is a large body of work that has identified roles of CP-AMPARs in hippocampal interneurons as well as in other forms of hippocampal synaptic plasticity. In addition, there is substantial evidence for roles of CP-AMPARs in synaptic plasticity at brain structures beyond the hippocampus, the coverage of which is outside of the scope of this review article. Clearly the field has progressed substantially since the first description of roles for CP-AMPARs in synaptic plasticity (Gu et al., 1996; Jia et al., 1996).

## Author Contributions

All authors contributed to the review article.

## Conflict of Interest Statement

The authors declare that the research was conducted in the absence of any commercial or financial relationships that could be construed as a potential conflict of interest.

## References

[B1] Abraham W. C. Otani S. (1991). “Macromolecules and the maintenance of long-term potentiation,” in Kindling and Synaptic Plasticity, ed. Morrell F. (Boston: Birkhäuser), 92–109.

[B2] Adesnik H. Nicoll R. A. (2007). Conservation of glutamate receptor 2-containing AMPA receptors during long-term potentiation. J. Neurosci. 27, 4598–4602. 10.1523/JNEUROSCI.0325-07.2007 17460072 PMC6672988

[B3] Asrar S. Zhou Z. Ren W. Jia Z. (2009). Ca^2+^ permeable AMPA receptor induced long-term potentiation requires PI3/MAP kinases but not Ca/CaM-dependent kinase II. PLoS One 4:e4339. 10.1371/journal.pone.0004339 19190753 PMC2629531

[B4] Barad M. Bourtchouladze R. Winder D. G. Golan H. Kandel E. (1998). Rolipram, a type IV-specific phosphodiesterase inhibitor, facilitates the establishment of long-lasting long-term potentiation and improves memory. Proc. Natl. Acad. Sci. U S A 95, 15020–15025. 10.1073/pnas.95.25.15020 9844008 PMC24568

[B5] Bashir Z. I. Bortolotto Z. A. Davies C. H. Berretta N. Irving A. J. Seal A. J.. (1993). Induction of LTP in the hippocampus needs synaptic activation of glutamate metabotropic receptors. Nature 363, 347–350. 10.1038/363347a0 8388549

[B6] Benke T. A. Lüthi A. Isaac J. T. R. Collingridge G. L. (1998). Modulation of AMPA receptor unitary conductance by synaptic activity. Nature 393, 793–797. 10.1038/31709 9655394

[B7] Bliss T. V. P. Collingridge G. L. (1993). A synaptic model of memory: long-term potentiation in the hippocampus. Nature 361, 31–39. 10.1038/361031a0 8421494

[B8] Bliss T. V. P. Collingridge G. L. Morris R. G. M. Reymann K. G. (2018). Long-term potentiation in the hippocampus: discovery, mechanisms and function. Neuroforum 24, A103–A120. 10.1515/nf-2017-a059

[B10] Bortolotto Z. A. Bashir Z. I. Davies C. H. Collingridge G. L. (1994). A molecular switch activated by metabotropic glutamate receptors regulates induction of long-term potentiation. Nature 368, 740–743. 10.1038/368740a0 8152485

[B9] Bortolotto Z. A. Collett V. J. Conquet F. Jia Z. van der Putten H. Collingridge G. L. (2005). The regulation of hippocampal LTP by the molecular switch, a form of metaplasticity, requires mGlu5 receptors. Neuropharmacology 49, 13–25. 10.1016/j.neuropharm.2005.05.020 16024054

[B11] Burnashev N. Monyer H. Seeburg P. H. Sakmann B. (1992). Divalent ion permeability of AMPA receptor channels is dominated by the edited form of a single subunit. Neuron 8, 189–198. 10.1016/0896-6273(92)90120-3 1370372

[B12] Clem R. L. Huganir R. L. (2010). Calcium-permeable AMPA receptor dynamics mediate fear memory erasure. Science 330, 1108–1112. 10.1126/science.1195298 21030604 PMC3001394

[B13] Cohen A. S. Raymond C. R. Abraham W. C. (1998). Priming of long-term potentiation induced by activation of metabotropic glutamate receptors coupled to phospholipase C. Hippocampus 8, 160–170. 10.1002/(sici)1098-1063(1998)8:2<160::aid-hipo8>3.0.co;2-p 9572722

[B14] Collingridge G. L. (1985). Long term potentiation in the hippocampus: mechanisms of initiation and modulation by neurotransmitters. Trends Pharmacol. Sci. 6, 407–411. 10.1016/0165-6147(85)90192-0

[B15] Collingridge G. L. Isaac J. T. R. Wang Y. T. (2004). Receptor trafficking and synaptic plasticity. Nat. Rev. Neurosci. 5, 952–962. 10.1038/nrn1556 15550950

[B16] Croce A. Pelletier J. G. Tartas M. Lacaille J.-C. (2010). Afferent-specific properties of interneuron synapses underlie selective long-term regulation of feedback inhibitory circuits in CA1 hippocampus. J. Physiol. 588, 2091–2107. 10.1113/jphysiol.2010.189316 20403974 PMC2911214

[B17] Davies S. N. Lester R. A. Reymann K. G. Collingridge G. L. (1989). Temporally distinct pre- and post-synaptic mechanisms maintain long-term potentiation. Nature 338, 500–503. 10.1038/338500a0 2564640

[B18] Dias R. B. Rombo D. M. Ribeiro J. A. Sebastião A. M. (2013). Ischemia-induced synaptic plasticity drives sustained expression of calcium-permeable AMPA receptors in the hippocampus. Neuropharmacology 65, 114–122. 10.1016/j.neuropharm.2012.09.016 23041538

[B19] Donevan S. D. Rogawski M. A. (1995). Intracellular polyamines mediate inward rectification of Ca^2+^-permeable α-amino-3-hydroxy-5-methyl-4-isoxazolepropionic acid receptors. Proc. Natl. Acad. Sci. U S A 92, 9298–9302. 10.1073/pnas.92.20.9298 7568121 PMC40972

[B20] Fortin D. A. Davare M. A. Srivastava T. Brady J. D. Nygaard S. Derkach V. A.. (2010). Long-term potentiation-dependent spine enlargement requires synaptic Ca^2+^-permeable AMPA receptors recruited by CaM-kinase I. J. Neurosci. 30, 11565–11575. 10.1523/JNEUROSCI.1746-10.2010 20810878 PMC2943838

[B21] Frey U. Huang Y. Y. Kandel E. R. (1993). Effects of cAMP simulate a late stage of LTP in hippocampal CA1 neurons. Science 260, 1661–1664. 10.1126/science.8389057 8389057

[B22] Gardner S. M. Takamiya K. Xia J. Suh J.-G. Johnson R. Yu S.. (2005). Calcium-permeable AMPA receptor plasticity is mediated by subunit-specific interactions with PICK1 and NSF. Neuron 45, 903–915. 10.1016/j.neuron.2005.02.026 15797551

[B23] Gray E. E. Fink A. E. Sarinana J. Vissel B. O’Dell T. J. (2007). Long-term potentiation in the hippocampal CA1 region does not require insertion and activation of GluR2-lacking AMPA receptors. J. Neurophysiol. 98, 2488–2492. 10.1152/jn.00473.2007 17652419

[B24] Gu J. G. Albuquerque C. Lee C. J. MacDermott A. B. (1996). Synaptic strengthening through activation of Ca^2+^-permeable AMPA receptors. Nature 381, 793–796. 10.1038/381793a0 8657283

[B25] Guire E. S. Oh M. C. Soderling T. R. Derkach V. A. (2008). Recruitment of calcium-permeable AMPA receptors during synaptic potentiation is regulated by CaM-kinase I. J. Neurosci. 28, 6000–6009. 10.1523/JNEUROSCI.0384-08.2008 18524905 PMC2671029

[B26] Hayashi Y. Shi S. H. Esteban J. A. Piccini A. Poncer J. C. Malinow R. (2000). Driving AMPA receptors into synapses by LTP and CaMKII: requirement for GluR1 and PDZ domain interaction. Science 287, 2262–2267. 10.1126/science.287.5461.2262 10731148

[B27] He K. Song L. Cummings L. W. Goldman J. Huganir R. L. Lee H.-K. (2009). Stabilization of Ca^2+^-permeable AMPA receptors at perisynaptic sites by GluR1–S845 phosphorylation. Proc. Natl. Acad. Sci. U S A 106, 20033–20038. 10.1073/pnas.0910338106 19892736 PMC2785287

[B28] Herron C. E. Lester R. A. Coan E. J. Collingridge G. L. (1986). Frequency-dependent involvement of NMDA receptors in the hippocampus: a novel synaptic mechanism. Nature 322, 265–268. 10.1038/322265a0 2874493

[B29] Huang Y. Y. Kandel E. R. (1994). Recruitment of long-lasting and protein kinase A-dependent long-term potentiation in the CA1 region of hippocampus requires repeated tetanization. Learn. Mem. 1, 74–82. 10467587

[B30] Isa T. Iino M. Itazawa S. Ozawa S. (1995). Spermine mediates inward rectification of Ca^2+^-permeable AMPA receptor channels. Neuroreport 6, 2045–2048. 10.1097/00001756-199510010-00022 8580437

[B31] Jaafari N. Henley J. M. Hanley J. G. (2012). PICK1 mediates transient synaptic expression of GluA2-lacking AMPA receptors during glycine-induced AMPA receptor trafficking. J. Neurosci. 32, 11618–11630. 10.1523/JNEUROSCI.5068-11.2012 22915106 PMC6703756

[B32] Jia Z. Agopyan N. Miu P. Xiong Z. Henderson J. Gerlai R.. (1996). Enhanced LTP in mice deficient in the AMPA receptor GluR2. Neuron 17, 945–956. 10.1016/s0896-6273(00)80225-1 8938126

[B33] Ju W. Morishita W. Tsui J. Gaietta G. Deerinck T. J. Adams S. R.. (2004). Activity-dependent regulation of dendritic synthesis and trafficking of AMPA receptors. Nat. Neurosci. 7, 244–253. 10.1038/nn1189 14770185

[B34] Kim S. Titcombe R. F. Zhang H. Khatri L. Girma H. K. Hofmann F.. (2015). Network compensation of cyclic GMP-dependent protein kinase II knockout in the hippocampus by Ca^2+^-permeable AMPA receptors. Proc. Natl. Acad. Sci. U S A 112, 3122–3127. 10.1073/pnas.1417498112 25713349 PMC4364185

[B35] Kim S. Ziff E. B. (2014). Calcineurin mediates synaptic scaling via synaptic trafficking of Ca^2+^-permeable AMPA receptors. PLoS Biol. 12:e1001900. 10.1371/journal.pbio.1001900 24983627 PMC4077568

[B36] Laezza F. Doherty J. J. Dingledine R. (1999). Long-term depression in hippocampal interneurons: joint requirement for pre- and postsynaptic events. Science 285, 1411–1414. 10.1126/science.285.5432.1411 10464102

[B37] Lei S. McBain C. J. (2002). Distinct NMDA receptors provide differential modes of transmission at mossy fiber-interneuron synapses. Neuron 33, 921–933. 10.1016/s0896-6273(02)00608-6 11906698

[B39] Liu S. J. Cull-Candy S. G. (2000). Synaptic activity at calcium-permeable AMPA receptors induces a switch in receptor subtype. Nature 405, 454–458. 10.1038/35013064 10839540

[B40] Liu S. J. Cull-Candy S. G. (2005). Subunit interaction with PICK and GRIP controls Ca^2+^ permeability of AMPARs at cerebellar synapses. Nat. Neurosci. 8, 768–775. 10.1038/nn1468 15895086

[B38] Liu B. Liao M. Mielke J. G. Ning K. Chen Y. Li L.. (2006). Ischemic insults direct glutamate receptor subunit 2-lacking AMPA receptors to synaptic sites. J. Neurosci. 26, 5309–5319. 10.1523/JNEUROSCI.0567-06.2006 16707783 PMC6675311

[B41] Lu Y. Allen M. Halt A. R. Weisenhaus M. Dallapiazza R. F. Hall D. D.. (2007). Age-dependent requirement of AKAP150-anchored PKA and GluR2-lacking AMPA receptors in LTP. EMBO J. 26, 4879–4890. 10.1038/sj.emboj.7601884 17972919 PMC2099463

[B42] Lu W.-Y. Man H.-Y. Ju W. Trimble W. S. MacDonald J. F. Wang Y. T. (2001). Activation of synaptic NMDA receptors induces membrane insertion of new AMPA receptors and LTP in cultured hippocampal neurons. Neuron 29, 243–254. 10.1016/s0896-6273(01)00194-5 11182095

[B43] Mainen Z. F. Jia Z. Roder J. Malinow R. (1998). Use-dependent AMPA receptor block in mice lacking GluR2 suggests postsynaptic site for LTP expression. Nat. Neurosci. 1, 579–586. 10.1038/2812 10196565

[B44] Makino H. Malinow R. (2009). AMPA receptor incorporation into synapses during LTP: the role of lateral movement and exocytosis. Neuron 64, 381–390. 10.1016/j.neuron.2009.08.035 19914186 PMC2999463

[B45] Malinow R. Malenka R. C. (2002). AMPA receptor trafficking and synaptic plasticity. Annu. Rev. Neurosci. 25, 103–126. 10.1146/annurev.neuro.25.112701.142758 12052905

[B46] Man H.-Y. Sekine-Aizawa Y. Huganir R. L. (2007). Regulation of α-amino-3-hydroxy-5-methyl-4-isoxazolepropionic acid receptor trafficking through PKA phosphorylation of the Glu receptor 1 subunit. Proc. Natl. Acad. Sci. U S A 104, 3579–3584. 10.1073/pnas.0611698104 17360685 PMC1805611

[B47] Matthies H. Reymann K. G. (1993). Protein kinase A inhibitors prevent the maintenance of hippocampal long-term potentiation. Neuroreport 4, 712–714. 10.1097/00001756-199306000-00028 8347813

[B48] Mattison H. A. Bagal A. A. Mohammadi M. Pulimood N. S. Reich C. G. Alger B. E.. (2014). Evidence of calcium-permeable AMPA receptors in dendritic spines of CA1 pyramidal neurons. J. Neurophysiol. 115, 263–275. 10.1152/jn.00578.2013 24760782 PMC4064414

[B49] McBain C. J. Dingledine R. (1993). Heterogeneity of synaptic glutamate receptors on CA3 stratum radiatum interneurones of rat hippocampus. J. Physiol. 462, 373–392. 10.1113/jphysiol.1993.sp019560 8101227 PMC1175306

[B50] Megill A. Tran T. Eldred K. Lee N. J. Wong P. C. Hoe H.-S.. (2015). Defective age-dependent metaplasticity in a mouse model of Alzheimer’s disease. J. Neurosci. 35, 11346–11357. 10.1523/jneurosci.5289-14.2015 26269641 PMC4532762

[B51] Morita D. Rah J.-C. Isaac J. T. R. (2014). Incorporation of inwardly rectifying AMPA receptors at silent synapses during hippocampal long-term potentiation. Philos. Trans. R. Soc. Lond. B Biol. Sci. 369:20130156. 10.1098/rstb.2013.0156 24298157 PMC3843887

[B52] Nishimune A. Isaac J. T. Molnar E. Noel J. Nash S. R. Tagaya M.. (1998). NSF binding to GluR2 regulates synaptic transmission. Neuron 21, 87–97. 10.1016/s0896-6273(00)80517-6 9697854

[B53] Noh K.-M. Yokota H. Mashiko T. Castillo P. E. Zukin R. S. Bennett M. V. L. (2005). Blockade of calcium-permeable AMPA receptors protects hippocampal neurons against global ischemia-induced death. Proc. Natl. Acad. Sci. U S A 102, 12230–12235. 10.1073/pnas.0505408102 16093311 PMC1189338

[B54] Nonaka M. Fitzpatrick R. Lapira J. Wheeler D. Spooner P. A. Corcoles-Parada M.. (2017). Everyday memory: towards a translationally effective method of modelling the encoding, forgetting and enhancement of memory. Eur. J. Neurosci. 46, 1937–1953. 10.1111/ejn.13637 28677201

[B55] Okada T. Yamada N. Kakegawa W. Tsuzuki K. Kawamura M. Nawa H.. (2001). Sindbis viral-mediated expression of Ca^2+^-permeable AMPA receptors at hippocampal CA1 synapses and induction of NMDA receptor-independent long-term potentiation. Eur. J. Neurosci. 13, 1635–1643. 10.1046/j.0953-816x.2001.01523.x 11328357

[B56] Oren I. Nissen W. Kullmann D. M. Somogyi P. Lamsa K. P. (2009). Role of ionotropic glutamate receptors in long-term potentiation in rat hippocampal CA1 oriens-lacunosum moleculare interneurons. J. Neurosci. 29, 939–950. 10.1523/JNEUROSCI.3251-08.2009 19176803 PMC2668821

[B57] Palmer M. J. Isaac J. T. R. Collingridge G. L. (2004). Multiple, developmentally regulated expression mechanisms of long-term potentiation at CA1 synapses. J. Neurosci. 24, 4903–4911. 10.1523/jneurosci.0170-04.2004 15163681 PMC6729367

[B58] Park P. Sanderson T. M. Amici M. Choi S.-L. Bortolotto Z. A. Zhuo M.. (2016). Calcium-permeable AMPA receptors mediate the induction of the protein kinase A-dependent component of long-term potentiation in the hippocampus. J. Neurosci. 36, 622–631. 10.1523/jneurosci.3625-15.2016 26758849 PMC4710778

[B59] Park P. Volianskis A. Sanderson T. M. Bortolotto Z. A. Jane D. E. Zhuo M.. (2014). NMDA receptor-dependent long-term potentiation comprises a family of temporally overlapping forms of synaptic plasticity that are induced by different patterns of stimulation. Philos. Trans. R. Soc. Lond. B Biol. Sci. 369:20130131. 10.1098/rstb.2013.0131 24298134 PMC3843864

[B60] Pellegrini-Giampietro D. E. Bennett M. V. Zukin R. S. (1992). Are Ca^2+^-permeable kainate/AMPA receptors more abundant in immature brain? Neurosci. Lett. 144, 65–69. 10.1016/0304-3940(92)90717-l 1331916

[B61] Pickard L. Noel J. Duckworth J. K. Fitzjohn S. M. Henley J. M. Collingridge G. L.. (2001). Transient synaptic activation of NMDA receptors leads to the insertion of native AMPA receptors at hippocampal neuronal plasma membranes. Neuropharmacology 41, 700–713. 10.1016/s0028-3908(01)00127-7 11640924

[B62] Pickard L. Noel J. Henley J. M. Collingridge G. L. Molnar E. (2000). Developmental changes in synaptic AMPA and NMDA receptor distribution and AMPA receptor subunit composition in living hippocampal neurons. J. Neurosci. 20, 7922–7931. 10.1523/jneurosci.20-21-07922.2000 11050112 PMC6772733

[B63] Plant K. Pelkey K. A. Bortolotto Z. A. Morita D. Terashima A. McBain C. J.. (2006). Transient incorporation of native GluR2-lacking AMPA receptors during hippocampal long-term potentiation. Nat. Neurosci. 9, 602–604. 10.1038/nn1678 16582904

[B64] Quintana P. Soto D. Poirot O. Zonouzi M. Kellenberger S. Muller D.. (2015). Acid-sensing ion channel 1a drives AMPA receptor plasticity following ischaemia and acidosis in hippocampal CA1 neurons. J. Physiol. 593, 4373–4386. 10.1113/JP270701 26174503 PMC4594240

[B65] Rajasekaran K. Todorovic M. Kapur J. (2012). Calcium-permeable AMPA receptors are expressed in a rodent model of status epilepticus. Ann. Neurol. 72, 91–102. 10.1002/ana.23570 22829271 PMC3408623

[B66] Raymond C. R. Thompson V. L. Tate W. P. Abraham W. C. (2000). Metabotropic glutamate receptors trigger homosynaptic protein synthesis to prolong long-term potentiation. J. Neurosci. 20, 969–976. 10.1523/jneurosci.20-03-00969.2000 10648701 PMC6774154

[B67] Reymann K. G. Frey J. U. (2007). The late maintenance of hippocampal LTP: requirements, phases, ‘synaptic tagging’, ‘late-associativity’ and implications. Neuropharmacology 52, 24–40. 10.1016/j.neuropharm.2006.07.026 16919684

[B68] Ross S. T. Soltesz I. (2001). Long-term plasticity in interneurons of the dentate gyrus. Proc. Natl. Acad. Sci. U S A 98, 8874–8879. 10.1073/pnas.141042398 11438685 PMC37528

[B69] Rozov A. Sprengel R. Seeburg P. H. (2012). GluA2-lacking AMPA receptors in hippocampal CA1 cell synapses: evidence from gene-targeted mice. Front. Mol. Neurosci. 5:22. 10.3389/fnmol.2012.00022 22375105 PMC3285882

[B70] Samoilova M. V. Buldakova S. L. Vorobjev V. S. Sharonova I. N. Magazanik L. G. (1999). The open channel blocking drug, IEM-1460, reveals functionally distinct α-amino-3-hydroxy-5-methyl-4-isoxazolepropionate receptors in rat brain neurons. Neuroscience 94, 261–268. 10.1016/s0306-4522(99)00326-7 10613516

[B71] Sanderson J. L. Gorski J. A. Dell’acqua M. L. (2016). NMDA receptor-dependent LTD requires transient synaptic incorporation of Ca^2+^-permeable AMPARs mediated by AKAP150-anchored PKA and calcineurin. Neuron 89, 1000–1015. 10.1016/j.neuron.2016.01.043 26938443 PMC4914360

[B72] Sanderson J. L. Scott J. D. Dell’acqua M. L. (2018). Control of homeostatic synaptic plasticity by AKAP-anchored kinase and phosphatase regulation of Ca^2+^-permeable AMPA receptors. J. Neurosci. 38, 2863–2876. 10.1523/JNEUROSCI.2362-17.2018 29440558 PMC5852664

[B73] Sans N. Vissel B. Petralia R. S. Wang Y.-X. Chang K. Royle G. A.. (2003). Aberrant formation of glutamate receptor complexes in hippocampal neurons of mice lacking the GluR2 AMPA receptor subunit. J. Neurosci. 23, 9367–9373. 10.1523/jneurosci.23-28-09367.2003 14561864 PMC6740562

[B74] Shimshek D. R. Jensen V. Celikel T. Geng Y. Schupp B. Bus T.. (2006). Forebrain-specific glutamate receptor B deletion impairs spatial memory but not hippocampal field long-term potentiation. J. Neurosci. 26, 8428–8440. 10.1523/jneurosci.5410-05.2006 16914668 PMC6674347

[B75] Soares C. Lee K. F. Nassrallah W. Béïque J. C. (2013). Differential subcellular targeting of glutamate receptor subtypes during homeostatic synaptic plasticity. J. Neurosci. 33, 13547–13559. 10.1523/jneurosci.1873-13.2013 23946413 PMC6705149

[B76] Stubblefield E. A. Benke T. A. (2010). Distinct AMPA-type glutamatergic synapses in developing rat CA1 hippocampus. J. Neurophysiol. 104, 1899–1912. 10.1152/jn.00099.2010 20685930 PMC2957466

[B77] Szabo A. Somogyi J. Cauli B. Lambolez B. Somogyi P. Lamsa K. P. (2012). Calcium-permeable AMPA receptors provide a common mechanism for LTP in glutamatergic synapses of distinct hippocampal interneuron types. J. Neurosci. 32, 6511–6516. 10.1523/jneurosci.0206-12.2012 22573673 PMC3355374

[B78] Terashima A. Cotton L. Dev K. K. Meyer G. Zaman S. Duprat F.. (2004). Regulation of synaptic strength and AMPA receptor subunit composition by PICK1. J. Neurosci. 24, 5381–5390. 10.1523/jneurosci.4378-03.2004 15190111 PMC3310907

[B79] Thiagarajan T. C. Lindskog M. Tsien R. W. (2005). Adaptation to synaptic inactivity in hippocampal neurons. Neuron 47, 725–737. 10.1016/j.neuron.2005.06.037 16129401

[B80] Tigaret C. M. Olivo V. Sadowski J. H. L. P. Ashby M. C. Mellor J. R. (2016). Coordinated activation of distinct Ca^2+^ sources and metabotropic glutamate receptors encodes Hebbian synaptic plasticity. Nat. Commun. 7:10289. 10.1038/ncomms10289 26758963 PMC4735496

[B81] Topolnik L. Congar P. Lacaille J.-C. (2005). Differential regulation of metabotropic glutamate receptor- and AMPA receptor-mediated dendritic Ca^2+^ signals by presynaptic and postsynaptic activity in hippocampal interneurons. J. Neurosci. 25, 990–1001. 10.1523/jneurosci.4388-04.2005 15673681 PMC6725617

[B82] Toth K. Suares G. Lawrence J. J. Philips-Tansey E. McBain C. J. (2000). Differential mechanisms of transmission at three types of mossy fiber synapse. J. Neurosci. 20, 8279–8289. 10.1523/jneurosci.20-22-08279.2000 11069934 PMC6773175

[B83] Volianskis A. Jensen M. S. (2003). Transient and sustained types of long-term potentiation in the CA1 area of the rat hippocampus. J. Physiol. 550, 459–492. 10.1113/jphysiol.2003.044214 12794181 PMC2343043

[B84] Volianskis A. Bannister N. Collett V. J. Irvine M. W. Monaghan D. T. Fitzjohn S. M.. (2013a). Different NMDA receptor subtypes mediate induction of long-term potentiation and two forms of short-term potentiation at CA1 synapses in rat hippocampus *in vitro*. J. Physiol. 591, 955–972. 10.1113/jphysiol.2012.247296 23230236 PMC3591708

[B85] Volianskis A. Collingridge G. L. Jensen M. S. (2013b). The roles of STP and LTP in synaptic encoding. PeerJ 1:e3. 10.7717/peerj.3 23638365 PMC3629019

[B86] Whitcomb D. J. Hogg E. L. Regan P. Piers T. Narayan P. Whitehead G.. (2015). Intracellular oligomeric amyloid-β rapidly regulates GluA1 subunit of AMPA receptor in the hippocampus. Sci. Rep. 5:10934. 10.1038/srep10934 26055072 PMC4460729

[B87] Whitehead G. Jo J. Hogg E. L. Piers T. Kim D.-H. Seaton G.. (2013). Acute stress causes rapid synaptic insertion of Ca^2+^ -permeable AMPA receptors to facilitate long-term potentiation in the hippocampus. Brain 136, 3753–3765. 10.1093/brain/awt293 24271563 PMC3859225

[B88] Wikström M. A. Matthews P. Roberts D. Collingridge G. L. Bortolotto Z. A. (2003). Parallel kinase cascades are involved in the induction of LTP at hippocampal CA1 synapses. Neuropharmacology 45, 828–836. 10.1016/s0028-3908(03)00336-8 14529720

[B89] Wiltgen B. J. Royle G. A. Gray E. E. Abdipranoto A. Thangthaeng N. Jacobs N.. (2010). A role for calcium-permeable AMPA receptors in synaptic plasticity and learning. PLoS One 5:e12818. 10.1371/journal.pone.0012818 20927382 PMC2947514

[B90] Yang Y. Wang X.-B. Zhou Q. (2010). Perisynaptic GluR2-lacking AMPA receptors control the reversibility of synaptic and spines modifications. Proc. Natl. Acad. Sci. U S A 107, 11999–12004. 10.1073/pnas.0913004107 20547835 PMC2900706

[B91] Zhou Q. Sheng M. (2013). NMDA receptors in nervous system diseases. Neuropharmacology 74, 69–75. 10.1016/j.neuropharm.2013.03.030 23583930

